# The Glucocorticoid Receptor Co‐Chaperone FKBP51 in the Adrenal Cortex Is Not Involved in Regulating Hypothalamic–Pituitary–Adrenal Activity in the Mouse

**DOI:** 10.1111/ejn.70213

**Published:** 2025-08-05

**Authors:** London Aman, Vera Sterlemann, Veronika Kovarova, Sowmya Narayan, Margherita Springer, Daniela Harbich, Bianca Schmid, Benjamin Jurek, Mathias V. Schmidt

**Affiliations:** ^1^ Research Group Neurobiology of Stress Resilience Max Planck Institute of Psychiatry Munich Germany; ^2^ Graduate School of Systemic Neurosciences, Ludwig Maximilians University (GSN‐LMU) Munich Germany; ^3^ International Max Planck Research School for Translational Psychiatry (IMPRS‐TP) Munich Germany; ^4^ Max Planck Institute of Psychiatry Munich Germany

**Keywords:** adrenal cortex, chronic social defeat, FKBP5, HPA axis, stress response

## Abstract

The FK506 binding protein 51 (FKBP51) is a stress‐modulating protein implicated in stress‐related psychiatric disorders. While FKBP51 has been extensively studied in the brain and other tissues, its role in the adrenal gland, a key part of the stress response, remains unexplored. To investigate *Fkbp5* gene expression and FKBP51 function in the adrenal gland, we examined expression in C57Bl/6 male and female mice and generated an adrenal cortex‐specific *Fkbp5* knockout (AS^*Fkbp5*−/−^). We found *Fkbp5* mRNA expression throughout the adrenal gland in both male and female mice. However, deletion of *Fkbp5* in the adrenal cortex did not alter basal stress hormone levels or acute restraint stress responses in both sexes and had no effect on chronic social defeat stress responses in male mice. These findings suggest that FKBP51 in the adrenal cortex plays another role than critically mediating acute or chronic social stress responses and highlight the potential for peripheral FKBP51 manipulations in the treatment of stress‐related disorders with no off‐target effects at the adrenal level.

## Graphical abstract

Despite the expression of FKBP51 throughout the adrenal cortex, its deletion did not alter HPA axis activity after acute or chronic stress in male and female mice. This finding suggests that adrenal FKBP51 is not a critical regulator of the stress response, highlighting its potential as a peripheral therapeutic target with minimal risk of adrenal off‐target effects.

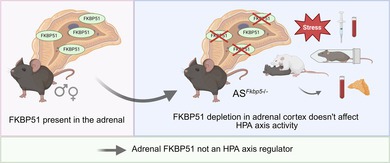

**Abbreviations**
ACTH: adrenocorticotropic hormone
ANOVA: analysis of variance
AS: aldosterone synthase
BBB: blood brain barrier
BNST: bed nucleus of the stria terminalis
CORT: corticosterone/cortisol
CRH: corticotropin‐releasing hormone
CSDS: chronic social defeat stress
DEX: dexamethasone
FKBP51: FK506 binding protein 51
GC: glucocorticoid
GR: glucocorticoid receptor
HPA Axis: hypothalamic–pituitary–adrenal Axis
HSP90: heat shock protein 90
MC2R: melanocortin 2 receptor
PVN: paraventricular nucleus
TH: tyrosine hydroxylase
zF: zona fasciculata
zG: zona glomerulosa

## 1 Introduction

Stress‐related psychiatric disorders, such as major depressive disorder, generalized anxiety disorder, or post‐traumatic stress disorder, affect the quality of life of millions worldwide diagnosed and represent a tremendous burden for our society (GBD 2019 Mental Disorders Collaborators 2022). These disorders often arise from an interplay between environmental challenges, such as traumatic or chronic stress exposure, and genetic predispositions that heighten the individual's stress susceptibility through a dysregulated hypothalamic–pituitary–adrenal (HPA) axis response (Matosin et al. 2018). One of these genetic factors that has emerged as a promising candidate explaining the mechanisms underlying these disorders is *Fkbp5*, encoding the co‐chaperone protein FK506‐binding protein 51 (FKBP51) (Fries et al. 2017). Polymorphisms in the *Fkbp5* gene that lead to heightened FKBP51 expression have repeatedly been associated with stress‐related psychiatric disorders (Criado‐Marrero et al. 2018).

As a co‐chaperone, FKBP51 modulates protein–protein interactions and can thereby affect protein signaling and function (Baischew et al. 2023; Häusl et al. 2019). FKBP51 interacts with the glucocorticoid receptor (GR), one of the main receptors for the stress hormone corticosterone (CORT, cortisol in humans), which is released following stress exposure through HPA axis activation (De Kloet et al. 2005). Besides the numerous physiological effects of GCs throughout the whole body, such as energy balance and glucose utilization, GCs cross the blood–brain barrier to activate the available GRs or mineralocorticoid receptors (MRs) (McEwen and Akil 2020). Acting as a repressor of GR function, FKBP51 presence in the GR‐HSP90 complex lowers ligand affinity and nuclear GR translocation (Noddings et al. 2023). This generates an ultra‐short feedback loop balancing FKBP51 expression and GR sensitivity within the stress response.

Previously demonstrated to be a critical regulator of the HPA axis across multiple species, FKBP51 has been found to directly affect HPA axis function in a region‐ and cell type‐specific manner (Schmidt et al. 2015). Specifically, FKBP51 in the paraventricular nucleus (PVN), the central starting point of the HPA axis, was identified to crucially shape HPA axis activity through modulation of GR (Häusl et al. 2021). Additionally, FKBP51 in brain regions upstream of the PVN, like the bed nucleus of the stria terminalis (BNST) (Engelhardt et al. 2021) or downstream in the HPA axis, like the anterior pituitary (Brix et al. 2022), was demonstrated to influence HPA axis regulation.

A crucial organ for stress regulation and HPA axis function is the adrenal, comprised of two distinct regions: the inner medulla and the outer cortex. Within the adrenal cortex, there are distinct zones, specifically the zona glomerulosa (zG) and the glucocorticoid‐producing zona fasciculata (zF) (Pignatti et al. 2017). Adrenal function has been linked to the dysregulation of the HPA axis and increased risk for psychiatric disorders (Pivonello et al. 2015). Alterations in adrenal size and function are classically used as indicators of HPA axis dysregulation within models of chronic stress, such as chronic social defeat stress (CSDS) in rodents (Van Doeselaar et al. 2021). Additionally, recent evidence from single‐cell RNA sequencing studies pointed to a significant expression of *Fkbp5* in the adrenal (Lopez et al. 2021), but its function there has not yet been elucidated.

Given the involvement of FKBP51 in glucocorticoid signaling, it is plausible that FKBP51 plays a significant role in adrenal function in response to acute and chronic stress. By investigating the expression and function of FKBP51 in the adrenal cortex utilizing C57Bl/6n mice and an adrenal cortex‐specific KO of *Fkbp5*, this study aims to shed light on the potential contribution of adrenal FKBP51 in the response to acute and chronic stress to HPA axis function and stress‐related disorders.

## 2 Methods

### 2.1 Animals and Animal Housing

All experiments and protocols were approved by the committee for the Care and Use of Laboratory animals of the Government of Upper Bavaria and were performed in accordance with the European Communities' Council Directive 2010/63/EU. Throughout the experiments, all effort was made to minimize any suffering of the animals. C57Bl/6n mice were bred in an in‐house colony through the Max Planck Institute of Psychiatry breeding facility, and the mouse lines *Fkbp5* ^lox/lox^ and *AS* ^*Fkbp5*−/−^ were generated in‐house on a C57Bl/6n background. Experiments were performed on animals aged 3–6 months at the onset of the experiments. All animals were kept group housed in individually ventilated cages (IVC; 30 cm × 16 cm × 16 cm; 501 cm^2^) serviced by a central airflow system (Tecniplast IVS Green Line—GM500). Animals were provided ad libitum access to water and food (standard research diet by Altromin 1318, Altromin GmbH, Germany) and were maintained under constant environmental conditions (12:12‐h light/dark cycle, 23°C ± 2°C, humidity of 55%). IVCs had sufficient bedding and nesting material as well as a wooden tunnel for environmental enrichment. Animals were allocated to experimental groups in a semirandomized fashion, and data analysis was performed blinded to group and genotype allocation.

### 2.2 Generation of *AS* ^*Fkbp5*−/−^ Line

Aldosterone synthase (AS) is expressed exclusively in fully differentiated cells of zG, which were shown to subsequently undergo lineage conversion into zF cells (Freedman et al. 2013). Mice lacking *Fkbp5* in the adrenal cortex were therefore obtained by breeding *Fkbp5* ^lox/lox^ mice (Häusl et al. 2021) to AS‐Cre mice (Lambert‐Langlais et al. 2009), resulting in *Fkbp5* ^lox/lox^ AS‐Cre (termed AS^*Fkbp5*−/−^) and their wild‐type *Fkbp5* ^lox/lox^ littermates. To genotype the *Fkbp5* alleles, primer 1, 5’‐ATGAAGGTCACGTGCTCAGG‐3’, was used to detect the wt allele (249 bp), primer 2, 5’‐AATAAAGCCTAGGACCCGCC‐3’, was used to detect the *lox* allele (456 bp), primer 3, 5’‐GGCGAGCTCAGACCATAACT‐3’ was used to detect the *del* (389 bp) and the *wt* (1239 bp) alleles, and primer 4, 5’‐ACTATCTCACAAGCCGTCCA‐3’, was utilized to detect all four. To genotype the cre transgene, two primers (5’‐CCACCAGCCAGCTATCAACT‐3′ and 5′‐CAGCTTGCATGATCTCCGGT‐3′) were utilized to amplify a *cre‐*specific product with (249 bp) or without (184 bp) a SV40 intron, as compared to a control product (372 bp) in which primers 5’‐TCTGAGTGGCAAAGGACCTTAGG‐3’ and 5’‐CCACTGGTGAGGTTGAGG‐3’ were used.

### 2.3 Experimental Design

To investigate the impact of *Fkbp5* knockout in the adrenal cortex on stress system biology, *AS* ^Fkbp5−/−^ mice (n_male_ = 5–6; n_female_ = 7–13) or *Fkbp5* ^lox/lox^ controls (n_male_ = 8–13; n_female_ = 7–13) were assessed under basal conditions for effects on body physiology and stress‐related endocrine phenotyping. A separate cohort of *AS* ^Fkbp5−/−^ (*n* = 8–13) and *Fkbp5* ^lox/lox^ (*n* = 9–13) male mice was challenged with 21 consecutive days of the CSDS paradigm. Prior to CSDS start, the response to an acute stressor was assessed for within‐animal comparison to post‐CSDS acute stress response. From day 16 until day 20 of the experimental period, HPA axis function and acute stress responsiveness were assessed. One day after the last defeat, all animals were sacrificed under basal conditions.

### 2.4 CSDS: Paradigm

Prior to the start of the CSDS experiment, resident CD1 males were tested on the likelihood to attack an intruder C57Bl/6n male, and the most aggressive CD1 mice were selected for the following CSDS experiment. The CSDS paradigm was performed as previously described, in which the experimental mouse is introduced to a resident CD1 mouse until social defeat is achieved, and then housed 24 h in the same cage separated by a divider (Wagner et al. 2011). This was repeated using a rotation schedule to present a novel resident to the stressed mouse each day of the 21 days of defeat. Fur status and body weight were determined every 3 days prior to the social defeat for all mice. The condition of the fur was assessed by an experienced investigator as described previously (Mineur et al. 2003). Summarized, fur status scores were classified according to a 4‐point scale, with perfect, clean fur scoring a 1 and disheveled, scruffy fur with possible traces of wounds scoring a 4. Respectively, scores of 2 and 3 were intermediate fur states.

### 2.5 Combined Dex/CRH Test

To investigate the negative feedback sensitivity of the HPA axis, we performed a combined dexamethasone (DEX)/corticotropin‐releasing hormone (CRH) test as described previously (Touma et al. 2011). In the morning (starting at 9 a.m.) of the experimental day, mice were injected i.p. with a low dose of DEX (0.05 mg/kg, Dex‐Ratiopharm, 7,633,932), a synthetic adrenal corticosteroid shown to predominantly act in the periphery and not cross the blood brain barrier (BBB) (Karssen et al. 2005). Six hours after DEX injection, blood was collected via tail cut (Fluttert et al. 2000), and mice were immediately injected i.p. with CRH (0.15 mg/kg, CRH Ferrin Amp). A second blood sample was obtained 30 min after CRH injection.

### 2.6 Acute Restraint Stress and CORT/ACTH Assessment

In the morning of the experimental day, starting at 9 a.m., animals were restrained in a 50‐mL Falcon tube for 30 min. Neuroendocrine profiles in response to acute restraint were obtained by tail cut blood samples 30 and 90 min following the onset of the restraint.

### 2.7 Blood Sampling and Hormone Measurements

All blood samples were collected in 1.5‐mL EDTA‐coated microcentrifuge tubes (Kabe Labortechnik, Germany), kept on ice, and centrifuged (8000 rpm, 15 min, at 4°C). At least 5 μL of plasma was collected and stored at −20°C for further processing. Corticosterone levels (ng/mL) and ACTH levels (pg/mL) were quantified by ELISA following the manufacturer's instructions (Corticosterone: Tecan, RE52211; ACTH: IBL international GmBH, RE53081).

### 2.8 Adrenal Sampling Procedure

At the day of sacrifice, animals were weighed, deeply anesthetized with isoflurane, and sacrificed by decapitation. Adrenals were dissected, pruned from fat, and either weighed or snap‐frozen in isopentane and stored at −80°C until further processing.

### 2.9 Knockout Validation of *Fkbp5* by RNAscope

The RNAscope Multiplex Fluorescent Reagent Kit (ACD Bio‐Techne) was used to stain for markers FKBP51 (mm‐Fkbp5‐C1), tyrosine hydroxylase (mm‐TH‐C2) and melanocortin 2 (mm‐MC2R‐C3). The *Fkbp5* probe spans Exon 9, which is deleted in our model, but also targets neighboring exons, resulting in possible binding to truncated mRNA. This may lead to a residual *Fkbp5* mRNA signal in knockout cells, even though no functional FKBP51 protein can be expressed. The staining procedure was performed as previously described, according to the manufacturer's specifications (Häusl et al. 2021). As no sex difference in our genetic strategy should be expected, imaging and quantification were performed on male samples. Images of the adrenal sections were obtained on a confocal microscope using a 20× or 40× objectives by an experimenter blinded to the condition of the animals. Settings for laser power, detector gain, and amplifier offset were identical for all images used for quantification. ImageJ (v1.54k) was utilized to analyze *Fkbp5* mRNA expression. *Fkbp5* mRNA puncta were counted manually in all AS+ cells per image (*n* = 20) and expressed as total *Fkbp5* puncta in AS+ cells. For determining the *Fkbp5* expression in the rest of the adrenal cortex, 30 AS− cells were manually selected in the DAPI channel at random, and *Fkbp5* mRNA puncta were counted and expressed as total *Fkbp5* puncta in AS− cells. All cells in the medulla were manually selected in the DAPI channel, and *Fkbp5* mRNA puncta were counted per cell. Due to the nature of the high expression of markers in the adrenal medulla and variance in visible tissue, expression was shown as the percent of medulla cells that were *Fkbp5* positive.

### 2.10 Statistical Analysis

All data were analyzed by GraphPad Prism 10.1.0 software (GraphPad Software, San Diego, California, USA). When two normally distributed groups were compared, the appropriate paired or unpaired student's *t* test was applied. In CSDS time‐course analysis of body weight and fur status, a repeated measures analysis of variance (ANOVA) with time as a within‐subject factor and genotype (AS^*Fkbp5−/−*^ vs. *Fkbp5* ^lox/lox^) as a between‐subject factor was applied. Due to the current study's main purpose of investigating the role of adrenal FKBP51, analyses of male and female cohorts were run and analyzed separately. Outliers were assessed with the GraphPad Prism outlier calculator performing the Grubb's two‐sided outlier test. *p* values of less than 0.05 were considered statistically significant. Data are shown as means ± SEM.

## 3 Results

### 3.1 *Fkbp5* Is Expressed Throughout the Male and Female C57Bl/6 Adrenal in a Region‐Specific Manner

**FIGURE 1:**
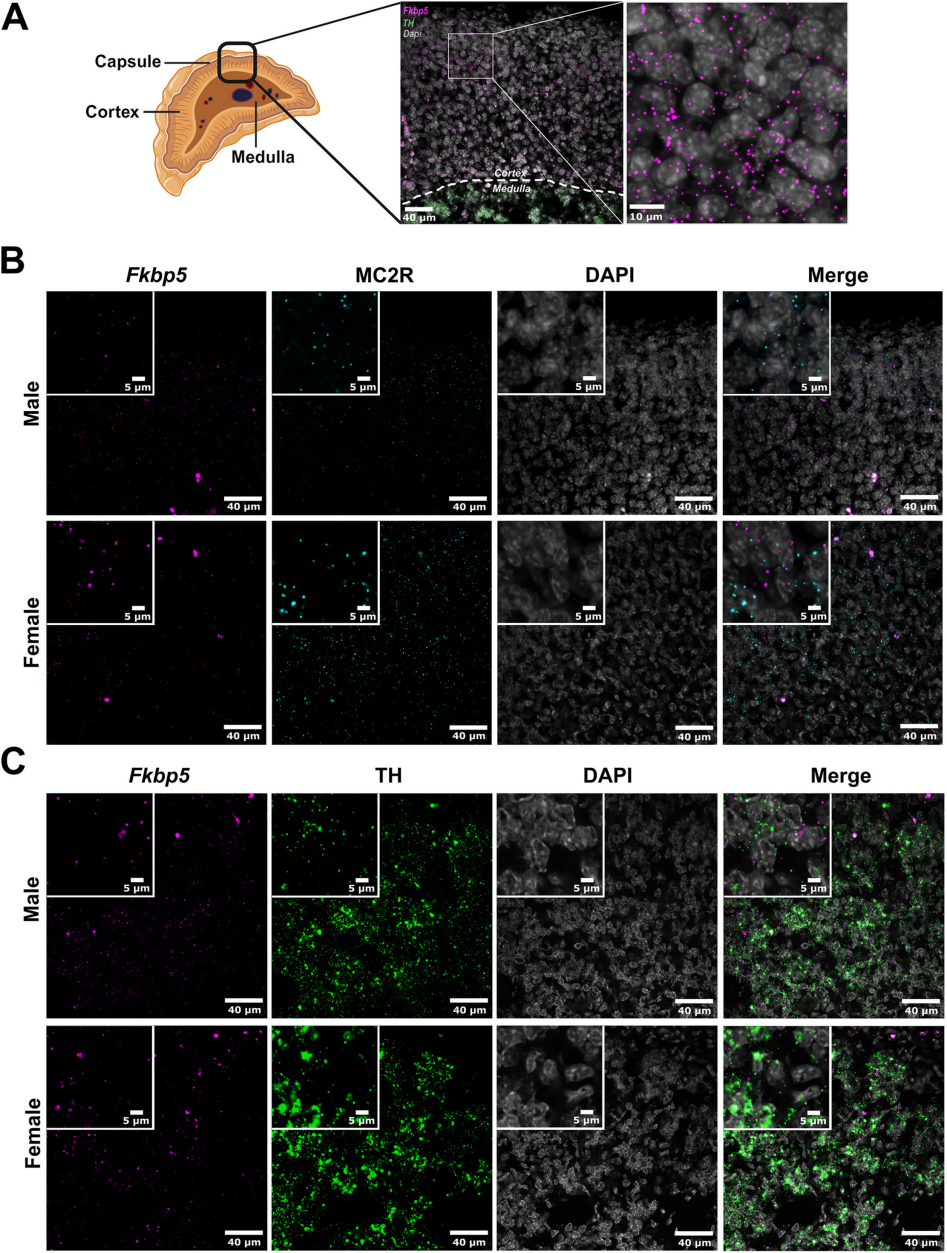
*Fkbp5* is expressed throughout the adrenal cortex in male and female mice. (A) Adrenal schematic and relative region shown in representative image, further zoomed in on right panel. (B) Representative confocal images of co‐expression of *Fkbp5* mRNA (pink) and zona fasciculata marker MC2R (cyan), DAPI (grey), and merged channels, with enlarged panel inserts of each image for detail. (C) Representative confocal images of co‐expression of *Fkbp5* mRNA (pink) and medulla marker TH (green), DAPI (grey), and merged channels, with enlarged panel inserts of each image for detail.

To understand basal *Fkbp5* expression patterns in the mouse adrenal, we imaged adrenal slices of C57Bl/6 male and female mice stained for *Fkbp5* mRNA along with other region‐specific markers to determine co‐expression patterns. Imaging and qualitative assessment across animals revealed *Fkbp5* (pink) was expressed in all zones of the adrenal, including the cortex, capsule, and medulla (Figure 1A). Co‐expression of *Fkbp5* and marker MC2R (cyan), encoding for the ACTH receptor in the zF of the adrenal cortex, was seen in male and female C57Bl/6 mice (Figure 1B; Walker et al. 2015). Similarly, co‐expression of *Fkbp5* and medulla marker TH (green) (Figure 1C; Bornstein et al. 2000) was also noted. Overall, *Fkbp5* expression was found in males and females in all regions of the adrenal.

### 3.2 *Fkbp5* Is Specifically Depleted in the Adrenal Cortex of AS^*Fkbp5−/−*^ Mice

**FIGURE 2:**
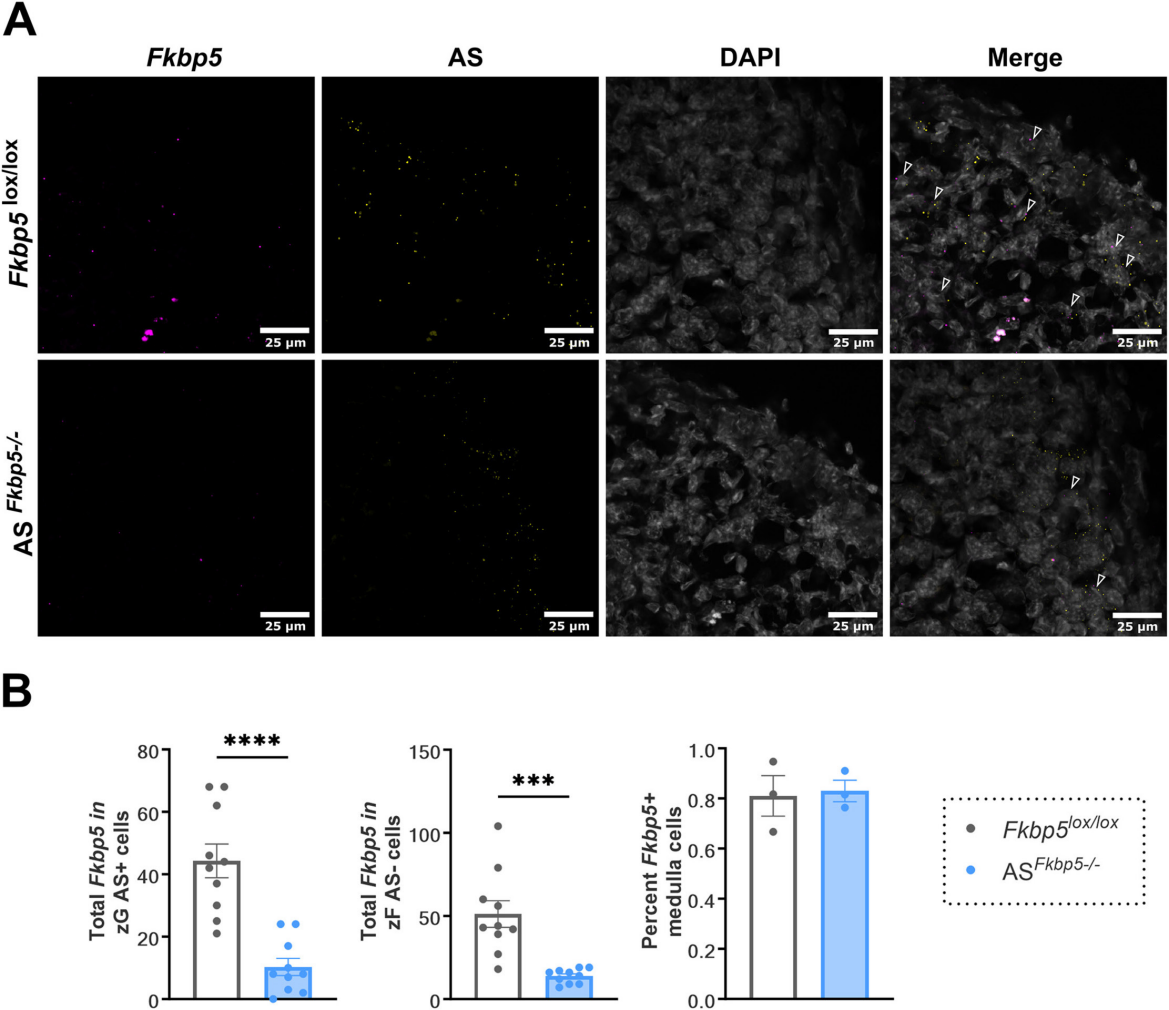
*Fkbp5* expression is depleted in conditional *Fkbp5* KO mice AS^*Fkbp5−/−*^. (A) Representative confocal images of *Fkbp5* mRNA (pink) and AS (yellow), DAPI (grey), and merged channels in *Fkbp5* ^lox/lox^ control and conditional KO AS^*Fkbp5−/−*^ mice. (B) Quantitative analysis of total *Fkbp5* puncta in confocal images in adrenal cortex (n_mice_ = 8, n_images_ = 10 vs. 10) AS+ cells and AS− cells revealed a signification reduction of *Fkbp5* in AS^*Fkbp5−/−*^ mice as compared to *Fkbp5* ^lox/lox^ control, with no difference of genotype seen in the medulla (n_mice_ = 4 n_images_ = 6). Data presented as mean ± SEM. ***p < 0.001, ****p < 0.0001.

To investigate the impact of FKBP51 disruption in the adrenal cortex, we generated conditional *Fkbp5* knockout mice targeting the glucocorticoid‐producing zF cells of the adrenal cortex (AS^*Fkbp5−/−*^). Aldosterone‐expressing cells undergo lineage conversion and subsequently lose their AS expression once converted to zF cells (Freedman et al. 2013). While now negative for AS, these cells in the zF that produce glucocorticoids were the main target of the conditional *Fkbp5* knockout. Therefore, it was critical to validate the genetic manipulation methods by assessing the depletion of *Fkbp5* mRNA in both currently AS+ cells and AS− cells. Successful reduction of *Fkbp5* expression (pink) was confirmed in AS^*Fkbp5−/−*^ mice as compared to control *Fkbp5* ^lox/lox^ mice (Figure 2A). Successful reduction of *Fkbp5* expression was assessed by quantitative analysis in AS (yellow) positive cells and AS negative cells compared to control *Fkbp5* ^lox/lox^ mice (AS+: t_18_ = 5.625, *p* < 0.0001, AS−: t_18_ = 4.631, *p* = 0.0002, n_mice_ = 8, n_images_ = 10 vs. 10). In addition, there was no difference in the expression of *Fkbp5* within the medulla, a region that should be untouched by the conditional knockout (t_4_ = 0.2149, *p* = 0.8403, n_mice_ = 4 n_images_ = 6). This validates that this line can be further utilized to investigate the effects of *Fkbp5* mRNA depletion and the role of FKBP51 in the adrenal cortex.

### 3.3 AS^*Fkbp5−/−*^ Male and Female Mice Do Not Differ in Baseline Stress‐Neuroendocrine Response or Acute Stress Response

**FIGURE 3:**
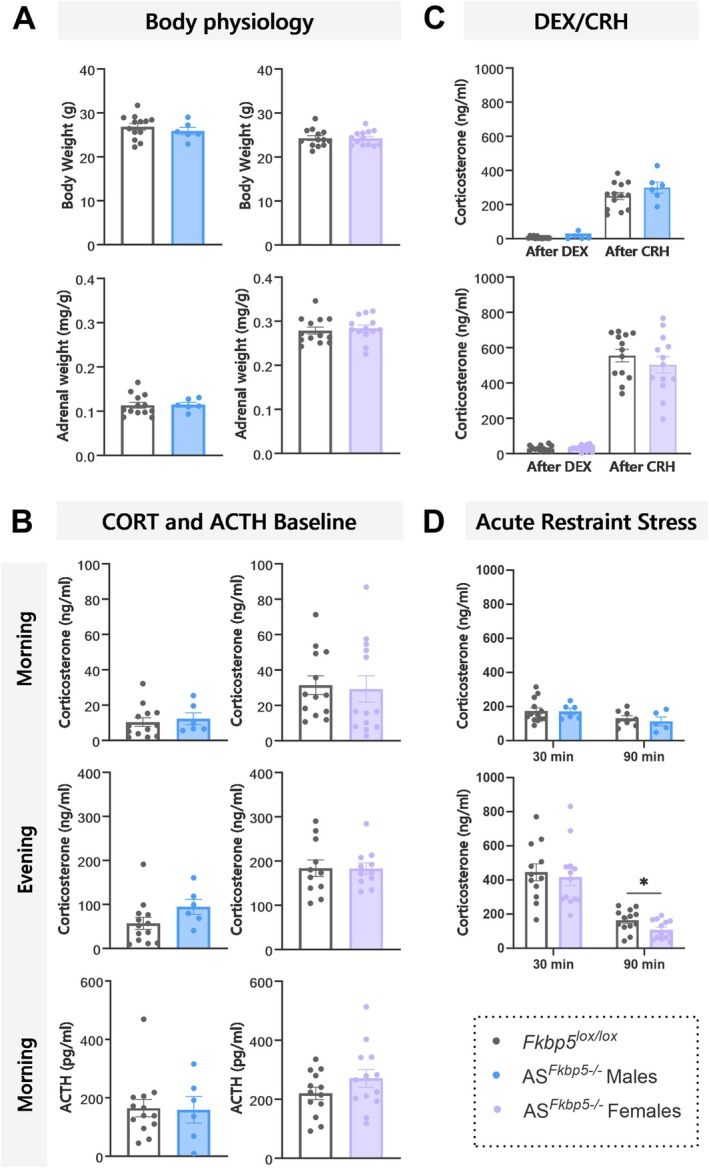
AS^*Fkbp5−/−*^ male and female mice do not differ from control at baseline. (A) Baseline body weight and relative adrenal weights in males and female AS^*Fkbp5−/−*^ (n_male_ = 5–6, n_female_ = 7–13) were unaltered compared to *Fkbp5* ^lox/lox^ control (n_male_ = 8–13, n_female_ = 7–13). (B) Baseline morning and evening CORT levels and morning ACTH levels were unaltered in AS^*Fkbp5−/−*^ mice. (C) In the Dex/CRH test, a similar significant CRH‐mediated CORT overshoot was observed in both AS^*Fkbp5−/−*^ and *Fkbp5* ^lox/lox^ mice, with no differences between genotypes observed. (D) Significantly decreased CORT levels were present in the recovery phase after the start of an acute restraint stress in both sexes in AS^*Fkbp5−/−*^ and control *Fkbp5* ^lox/lox^ animals. While no differences were observed in AS^*Fkbp5−/−*^ males compared to control, there was a slightly stronger decrease in CORT at 90 min observed in female AS^*Fkbp5−/−*^ as compared to control. Data presented as mean ± SEM. *p < 0.05.

To understand the impact of FKBP51 disruption on basal stress system biology, stress‐related endocrine phenotyping was performed on male and female AS^*Fkbp5−/−*^ and control *Fkbp5* ^lox/lox^ mice. Body weight (t_17_ = 0.7761, *p* = 0.4483) and adrenal weight (t_17_ = 0.1039, *p* = 0.9185) at the day of sampling did not differ in males, nor did body weight (t_24_ = 0.05385, *p* = 0.9575) and adrenal weight (t_24_ = 0.4154, *p* = 0.6815) differ in females (Figure 3A). No changes were observed in male baseline morning (9:00 a.m.) and evening (6:00 p.m.) CORT (morning: t_17_ = 0.4174, *p* = 0.6816, evening: t_17_ = 1.600, *p* = 0.1281) or female CORT (morning: t_24_ = 0.2348, *p* = 0.8164, evening: t_20_ = 0.03555, *p* = 0.9720) (Figure 3B). Morning ACTH (9:00 a.m.) did not differ in AS^*Fkbp5−/−*^ compared to control for males (t_16_ = 0.5024, *p* = 0.6223) or females (t_24_ = 1.378, *p* = 0.1809) (Figure 3B).

A significant increase in corticosterone was found after exposure to CRH following DEX in *Fkbp5* ^lox/lox^ control males (t_12_ = 11.46, *p* < 0.0001, *n* = 13) and females (t_12_ = 15.27, *p* < 0.0001, *n* = 13). Similarly, CRH‐mediated CORT levels also were significantly increased in AS^*Fkbp5−/−*^ males (t_4_ = 8.581, *p* = 0.0010, *n* = 5) and females (t_12_ = 10.66, *p* < 0.0001, *n* = 13), but no genotypic difference was observed at baseline in male Dex response (t_16_ = 1.307, *p* = 0.2096) or CRH response (t_17_ = 1.282, *p* = 0.2171) (Figure 3C). The same was observed in AS^*Fkbp5−/−*^ female DEX response (t_24_ = 0.1568, *p* = 0.8767) and CRH response (t_24_ = 0.8914, *p* = 0.3815) (Figure 3C).

We further assessed CORT response 30 and 90 min after an acute restraint stress, which revealed a significant drop in CORT levels during the stress recovery phase in AS^*Fkbp5−/−*^ males (t_4_ = 5.261, *p* = 0.0062), but no genotypic difference in males at either timepoint (30 min: t_17_ = 0.07073, *p* = 0.9444; 90 min: t_11_ = 0.6026, *p* = 0.5590). Investigating females revealed a significant drop in CORT levels during the stress recovery phase in AS^*Fkbp5−/−*^ females (t_12_ = 5.667, *p* < 0.0001) and in *Fkbp5* ^lox/lox^ control females (t_6_ = 3.381, *p* = 0.0148), but no genotypic difference in females at the earlier timepoint (30 min: t_18_ = 0.008752, *p* = 0.9931). Intriguingly, only after 90 min was a genotypic difference observed (t_19_ = 2.172, *p* = 0.0427). Overall, except for this one later timepoint in females after an acute stress, no major effects in male or female AS^*Fkbp5−/−*^ mice were observed under basal conditions.

### 3.4 Chronic Stress Does Not Differentially Affect Male AS^*Fkbp5−/−*^ Mice Compared to Controls

**FIGURE 4:**
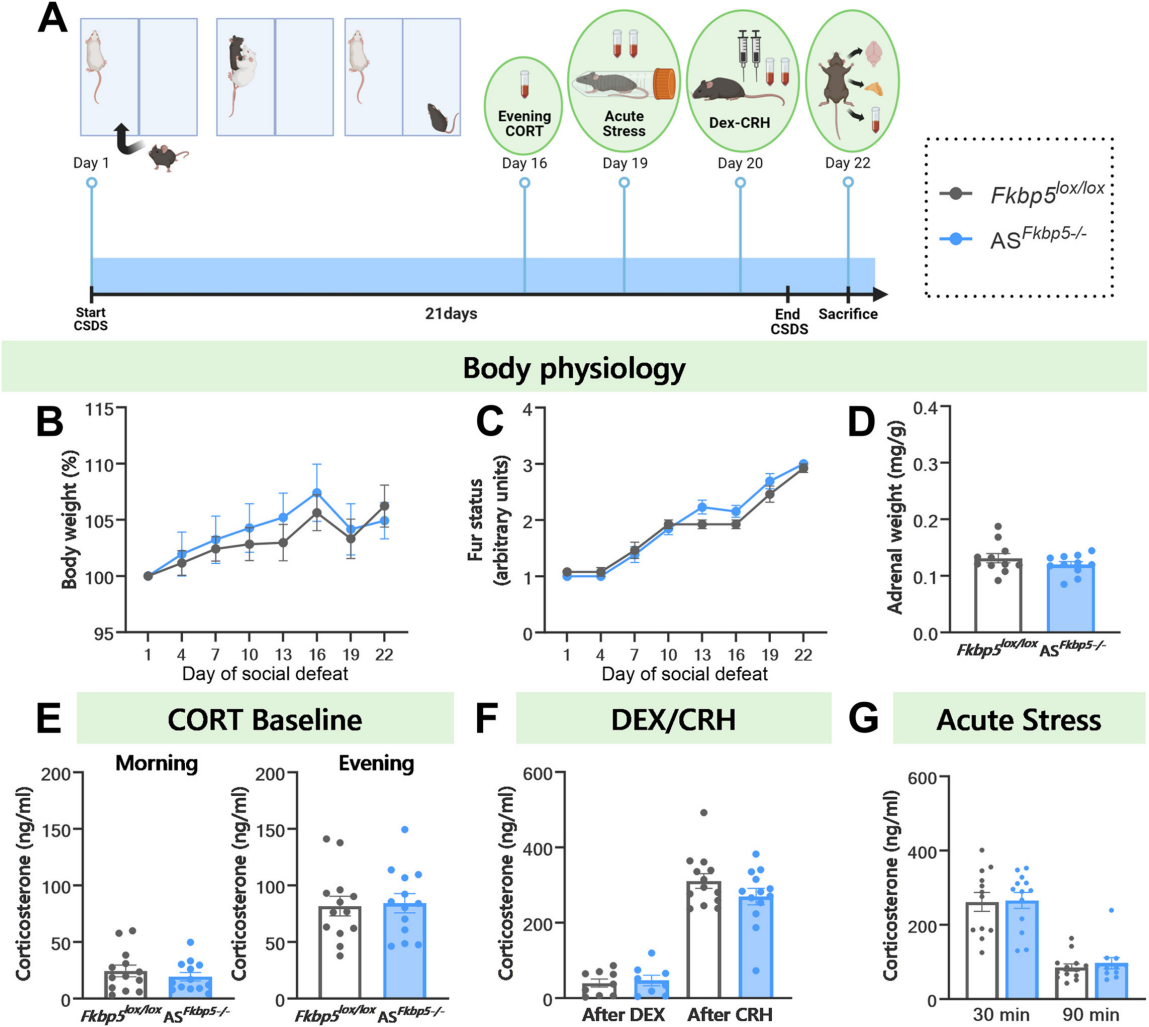
AS^*Fkbp5−/−*^ male mice do not differ from control under chronic social defeat stress. (A) Chronic social defeat stress for 21‐day protocol schematic (n_AS*Fkbp5*_^*−/−*^ = 8–13, n_*Fkbp5*_^lox/lox^ = 9–13). (B) No genotypic differences were found in chronic stress‐induced body weight increase or in (C) stress‐induced worsening of fur status. (D) Adrenal weights after CSDS were not different from control. (E) Baseline morning and evening CORT levels were unaltered in AS^*Fkbp5−/−*^ mice. (F) In the Dex/CRH test, a similar significant CRH‐mediated CORT overshoot was observed in both AS^*Fkbp5−/−*^ and *Fkbp5* ^lox/lox^ animals, with no differences between genotypes observed after chronic stress. (G) CORT levels significantly decreased at 90 min as compared to the 30 min timepoint in all animals after acute stress, with no genotypic differences seen. Data presented as mean ± SEM.

To further investigate the role of *Fkbp5* in the adrenal cortex in response to chronic stress, a longitudinal CSDS experiment was performed on AS^*Fkbp5−/−*^ male mice and their wild‐type littermates (Figure 4A). Before the start of the CSDS, we performed a restraint stress and assessed CORT response at 30 and 90 min, confirming the results of the first cohort of no genotypic differences at baseline (30 min: *Fkbp5* ^lox/lox^: 265.5 ± 17.11; AS^*Fkbp5−/−*^: 275.8 ± 15.98; t_24_ = 0.4424, *p* = 0.6621, 90 min: *Fkbp5* ^lox/lox^: 120.1 ± 16.47; AS^*Fkbp5−/−*^: 119.7 ± 21.52; t_20_ = 0.01358, *p* = 0.9893). At the beginning of the experiment, there was no difference in body weight between genotypes (AS^*Fkbp5−/−*^: 30.56 ± 0.7438, *Fkbp5* ^lox/lox^: 31.20 ± 0.9459, t_24_ = 0.5389, *p* = 0.5949).

For body weight throughout the CSDS, a repeated measures ANOVA revealed a significant within‐subjects effect for time (F_(2.501,47.52)_ = 12.86, *p* < 0.0001), but no time × genotype interaction (F_(7,133)_ = 0.5172, *p* = 0.8203), nor an in‐between‐subjects effect for genotype (F_(1,19)_ = 0.2865, *p* = 0.5987) (Figure 4B). For fur status throughout the CSDS, a repeated measures ANOVA revealed a significant within‐subjects effect for time (F_(3.808,91.40)_ = 123.5, *p* < 0.0001), but no time × genotype interaction (F_(7,168)_ = 1.795, *p* = 0.0912), nor an in‐between‐subjects effect for genotype (F_(1,24)_ = 0.8522, *p* = 0.3651) (Figure 4C).

Adrenal weight did not differ in AS^*Fkbp5−/−*^ mice compared to *Fkbp5* ^lox/lox^ control after 21 days of CSDS (t_20_ = 1.179, *p* = 0.2521) (Figure 4D). No differences were observed in AS^*Fkbp5−/−*^ morning (9:00 a.m.) and evening (6:00 p.m.) CORT after CSDS when compared to *Fkbp5* ^lox/lox^ control (morning: t_24_ = 0.8013, *p* = 0.4308; evening: t_24_ = 0.2030, *p* = 0.8409) (Figure 4E). A significant increase in corticosterone was found after exposure to CRH following DEX in AS^*Fkbp5−/−*^ (t_8_ = 8.237, *p* < 0.0001, *n* = 9) and *Fkbp5* ^lox/lox^ control (t_8_ = 10.93, *p* < 0.0001, *n* = 9) (Figure 4F). No difference between genotypes was observed at baseline in male Dex response (t_15_ = 0.4025, *p* = 0.6930) or CRH response (t_24_ = 1.414, *p* = 0.1702) (Figure 4F).

We further assessed CORT responses 30 and 90 min after an acute restraint stress, which revealed a significant drop in CORT levels during the stress recovery phase in AS^*Fkbp5−/−*^ (t_9_ = 8.173, *p* < 0.0001) and *Fkbp5* ^lox/lox^ control (t_11_ = 5.498, *p* = 0.0002), but no difference between genotypes at either timepoint (30 min: t_23_ = 0.1235, *p* = 0.9028; 90 min: t_21_ = 0.2518, *p* = 0.8037). Overall, no major differences in male AS^*Fkbp5−/−*^ mice were observed under CSDS conditions.

## 4 Discussion

In this study, new insights are provided into the expression profile and functional role of FKBP51 within the adrenal gland across both sexes. Results demonstrate that FKBP51 is expressed throughout the adrenal cortex and medulla of male and female C57Bl/6n mice. However, the selective deletion of FKBP51 in the adrenal cortex did not significantly impact stress‐related neuroendocrine responses under baseline conditions, nor did it alter responses following acute or chronic stress exposure.

While previous studies have explored the role of FKBP51 in the brain and other tissues (Brix et al. 2022; Häusl et al. 2021; Van Doeselaar et al. 2023), this investigation is the first to comprehensively characterize its expression patterns and potential stress‐related function within the adrenal gland. This, in addition to the adrenal's pivotal role in the stress response, led to reported acute and chronic stress challenges in a conditional adrenal cortex *Fkbp5*‐depleted mouse model.

The observed regional differences in FKBP51 expression suggest that FKBP51 may serve unique roles within the developmentally distinct adrenal medulla and the zG and zF of the mouse cortex. Given the established role of FKBP51 in modulating GR sensitivity, its presence in the zF—the primary site of glucocorticoid production but also a GR‐expressing target site of GCs—may have been anticipated to influence adrenal function, HPA axis regulation, or metabolic outcomes. Yet the lack of observable effects in adrenal‐specific FKBP51 knockout mice implies that FKBP51 may act independently of the GR in other adrenal processes. The effect of FKBP51 on GR function has been reported in multiple cell types and organs, without an indication that these effects are cell type or organ specific; therefore, while not explicitly measured in this study, it is unlikely that FKBP51 has another adrenal‐specific effect on GR function. Alternatively, the lack of FKBP51 in cells may exert subtle effects in the glucocorticoid‐producing cells of the zF, which could be masked by compensatory mechanisms within other GC‐responsive tissues or organs. Furthermore, stress‐responsive cell types in the adrenal, like macrophages or endothelial cells, might additionally modulate any effect (Lopez et al. 2021). It is possible that FKBP51 depletion in the zF influences physiological parameters of adrenal functions that were not assessed in our study. Nevertheless, given that our stress paradigm was based on previous findings that showed behavioral and cellular changes, as well as differentially expressed steroidogenic and *Fkbp5*‐related genes in the adrenal cortex after CSDS (Lopez et al. 2021), this is unlikely. Moreover, FKBP51 expressed in the hypothalamus and pituitary might potentially compensate, balancing the HPA axis response and mitigating potential behavioral or physiological outcomes. However, we did not detect significantly altered levels of CORT or ACTH that could compensate for a dysregulated adrenal functioning.

These regional differences in FKBP51 expression across adrenal zones may reflect potential divergence in function between the medulla and cortex, with FKBP51 possibly contributing differently in stress modulation within each zone. Notably, while GR sensitivity is typically associated with FKBP51 in the zF, the anticipated impact on GC release and further effects were not observed. FKBP51 expression in the medulla could signify compensatory mechanisms as well, as cells within the adrenal zones communicate and adapt adrenal function to different situations by influencing each other through regulatory circuits (Ehrhart‐Bornstein et al. 1998). Additionally, FKBP51 expression in the adrenal medulla was seen to be consistently stronger and more widespread than in the zF. A perceived stressor leads to the release of ACTH and therefore cortisol but also simultaneously activates the sympathetic nervous system and releases adrenaline and noradrenaline from the medulla. One could speculate FKBP51's expression in this region could indicate a parallel role of modulation, similar to FKBP51's role in modulating GR sensitivity, as cortical and medullary cells have been found to be interwoven for paracrine interactions and communication (Bechmann et al. 2021; Ehrhart‐Bornstem et al. 1995).

Several limitations should be acknowledged in this attempt to characterize FKBP51 function in the adrenal. This study's chronic stress paradigm was limited to male mice, and it is possible that females may exhibit different responses, especially as under basal conditions, we observed a slightly enhanced negative feedback to an acute stressor in females, but not males. Additionally, the genetic strategy used for *Fkbp5* deletion only introduces a step‐wise deletion of *Fkbp5* in the target cell population that migrates to the zF, which, although never described before in this context, may have introduced compensatory adaptations. To address these limitations, future studies might employ alternative genetic or pharmacological approaches, such as site‐directed CRISPR‐Cas, 3rd generation Gapmer LNA antisense oligos, the pharmacological FKBP51 inhibitor SAFit2 (Pöhlmann et al. 2018), or a FKBP51 specific proteolysis targeting chimera (PROTAC, Geiger et al. 2024), to better examine FKBP51's role in both male and female mice.

Overall, this study provides promising insights into the expression and possible function of FKBP51 within the adrenal gland. Although our findings suggest its role may not be central to mediating the stress response, further detailed analyses are essential to fully understand its interplay with the adrenal's GC synthesis and secretion. These insights highlight the potential for hypothalamic or pituitary FKBP51 targeting in therapeutic strategies for stress‐related disorders, without introducing off‐target effects at the level of the HPA axis executor: the adrenal.

## Author Contributions

**London Aman:** data curation, formal analysis, investigation, visualization, writing – original draft. **Vera Sterlemann:** conceptualization, data curation, formal analysis, investigation, writing – original draft. **Veronika Kovarova:** formal analysis, methodology, writing – review and editing. **Margherita Springer:** investigation, writing – review and editing. **Daniela Harbich:** investigation, writing – review and editing. **Bianca Schmid:** investigation. **Benjamin Jurek:** data curation, formal analysis, supervision, writing – review and editing. **Mathias V. Schmidt:** conceptualization, project administration, resources, supervision, writing – review and editing.

## Conflicts of Interest

The authors declare no conflicts of interest.

## Peer Review

The peer review history for this article is available at https://www.webofscience.com/api/gateway/wos/peer‐review/10.1111/ejn.70213.

## Data Availability

The authors confirm that the data supporting the findings of this study are available within the article. Raw data are available on request.
